# Growth-rate dependency of de novo resveratrol production in chemostat cultures of an engineered *Saccharomyces cerevisiae* strain

**DOI:** 10.1186/s12934-015-0321-6

**Published:** 2015-09-14

**Authors:** Tim Vos, Pilar de la Torre Cortés, Walter M. van Gulik, Jack T. Pronk, Pascale Daran-Lapujade

**Affiliations:** Department of Biotechnology, Delft University of Technology, Julianalaan 67, 2628 BC Delft, The Netherlands

**Keywords:** Metabolic engineering, Maintenance energy, Anabolic products, q_p_, Continuous culture, Yeast, Synthetic biology

## Abstract

**Introduction:**

*Saccharomyces cerevisiae* has become a popular host for production of non-native compounds. The metabolic pathways involved generally require a net input of energy. To maximize the ATP yield on sugar in *S. cerevisiae*, industrial cultivation is typically performed in aerobic, sugar-limited fed-batch reactors which, due to constraints in oxygen transfer and cooling capacities, have to be operated at low specific growth rates. Because intracellular levels of key metabolites are growth-rate dependent, slow growth can significantly affect biomass-specific productivity. Using an engineered *Saccharomyces cerevisiae* strain expressing a heterologous pathway for resveratrol production as a model energy-requiring product, the impact of specific growth rate on yeast physiology and productivity was investigated in aerobic, glucose-limited chemostat cultures.

**Results:**

Stoichiometric analysis revealed that de novo resveratrol production from glucose requires 13 moles of ATP per mole of produced resveratrol. The biomass-specific production rate of resveratrol showed a strong positive correlation with the specific growth rate. At low growth rates a substantial fraction of the carbon source was invested in cellular maintenance-energy requirements (e.g. 27 % at 0.03 h^−1^). This distribution of resources was unaffected by resveratrol production. Formation of the by-products coumaric, phloretic and cinnamic acid had no detectable effect on maintenance energy requirement and yeast physiology in chemostat. Expression of the heterologous pathway led to marked differences in transcript levels in the resveratrol-producing strain, including increased expression levels of genes involved in pathways for precursor supply (e.g. *ARO7* and *ARO9* involved in phenylalanine biosynthesis). The observed strong differential expression of many glucose-responsive genes in the resveratrol producer as compared to a congenic reference strain could be explained from higher residual glucose concentrations and higher relative growth rates in cultures of the resveratrol producer.

**Conclusions:**

De novo resveratrol production by engineered *S. cerevisiae* is an energy demanding process. Resveratrol production by an engineered strain exhibited a strong correlation with specific growth rate. Since industrial production in fed-batch reactors typically involves low specific growth rates, this study emphasizes the need for uncoupling growth and product formation via energy-requiring pathways.

**Electronic supplementary material:**

The online version of this article (doi:10.1186/s12934-015-0321-6) contains supplementary material, which is available to authorized users.

## Background

The budding yeast *Saccharomyces cerevisiae* is intensively used for metabolic engineering studies aimed at the production of non-native low-molecular compounds. In such research, the rapidly expanding toolbox for yeast synthetic biology is used for functional expression of heterologous product pathways, optimization of precursor supply from central carbon metabolism, minimization of by-product formation and efficient product export [1]. For successful implementation of engineered yeast strains in large-scale processes, energetics of product formation and conditions in industrial bioreactors need to be taken into consideration.

Virtually all non-native compounds produced by engineered *S. cerevisiae* strains require a net input of ATP for their formation from glucose [2–4]. In such scenarios, product formation competes for precursors and ATP with growth and maintenance processes [5]. In *S. cerevisiae*, the ATP yield from alcoholic fermentation is 2 mol (mol glucose)^−1^. The ATP yield from oxidative phosphorylation is determined by the P/O ratio: the number of ATP molecules synthesized for each electron pair transferred to oxygen via the mitochondrial respiratory chain [6]. Although the in vivo P/O ratio for oxidation of NADH and FADH in *S. cerevisiae* (ca. 1.0 [6]) is lower than in many other eukaryotes, respiratory glucose dissimilation still yields approximately 8-fold more ATP per mole of glucose than alcoholic fermentation. For yeast-based production of compounds whose synthesis requires a net input of ATP, it is therefore crucial that glucose dissimilation occurs exclusively via respiration.

Even under fully aerobic conditions, *S. cerevisiae* exhibits a predominantly fermentative metabolism when grown at high sugar concentrations [7]. Only at low to intermediate specific growth rates in aerobic, sugar-limited cultures, sugar dissimilation occurs exclusively via respiration. In industry, aerobic, sugar-limited yeast cultivation is typically performed in fed-batch reactors [8], in which the sugar feed rate controls the specific growth rate. However, the limited oxygen-transfer capacity and cooling capacity of large-scale (50–200 m^3^) bioreactors forces operators to decrease the specific growth rate when the dissolved oxygen concentration in bioreactors decreases to a critical value to prevent glucose dissimilation through alcoholic fermentation. Especially towards the end of high-biomass density fed-batch processes, this measure can result in specific growth rates that are below 5 % of the maximum specific growth rate observed in batch cultures grown on excess sugar [9, 10]. Therefore, prediction of the performance of strains in industrial processes requires quantitative data on growth-rate-dependent product formation. Ideally, performance under industrial conditions should already be taken into account in strain design and construction.

The relationship between specific growth rate (μ, h^−1^) and the biomass-specific rate of product formation (q_p_, mmol product (g biomass)^−1^ h^−1^) can be investigated in steady-state chemostat cultures, in which the specific growth rate equals the dilution rate [11]. Using this approach, a positive correlation between growth and product formation was found for several heterologous proteins [12, 13]. In the case of heterologous proteins, such a positive correlation of q_p_ and μ may be caused by several factors, including the capacity of the ribosomal machinery, size of amino-acyl-tRNA pools, activity of excretion pathways and cellular energy status. Unlike catabolic products, the formation of ATP-requiring products is not stoichiometrically coupled to growth. Instead, the distribution of carbon to either biomass or product formation depends on the competition between enzymes involved in anabolic routes and in the product synthetic pathway for precursors, ATP and co-factors. The sensitivity of such kinetics to changes in growth rate depends on a multitude of factors, in particular the nature of the synthetic route of the product of interest, the cellular concentration of key metabolites and the abundance and kinetic properties of the competing enzymes. The impact of growth on formation of an “anabolic” product is therefore extremely arduous to predict. So far, very few published studies describe the growth-rate dependency of physiological and production characteristics of non-native, ATP-requiring products in *S. cerevisiae* [14, 15].

Resveratrol (trans-3,5,4′-trihydroxystilbene) is a polyphenolic stilbenoid sold as neutraceutical and food ingredient. Reported health benefits include anti-oxidant effects, life span extension, inhibiting obesity and cancer prevention [16]. Commercial production of resveratrol from plant sources such as *Polygonum cuspidatum* is complicated by slow growth, low product yield, inconsistent performance, and difficult purification procedures [17]. Hence, the use of microbial production hosts has gained attention as a promising industrially relevant alternative. Formation of resveratrol from l-phenylalanine by engineered *S. cerevisiae* involves four heterologous reactions, catalysed by phenylalanine ammonia lyase (*PAL*) [18], cinnamate 4-hydroxylyase (*C4H*) [19] which associates with a heterologous cytochrome p450 reductase (*ATR2*) [20] and a native cytochrome b5 electron carrier (*CYB5*), 4-coumarate-CoA ligase (*4CL*) [21], and stilbene synthase (VST or STS) [4]. The latter enzyme reaction requires three malonyl-CoA molecules to form one molecule of resveratrol. Pathway stoichiometry predicts that de novo synthesis of resveratrol by the engineered yeast strain costs 12 mol ATP (mol resveratrol)^−1^, not taking into account possible ATP costs for product export or regeneration of co-factors, thereby making resveratrol a relevant model for an ATP-required, heterologous product of engineered *S. cerevisiae*.

Hitherto, studies on microbial production of resveratrol have focussed on metabolic pathway engineering in *Escherichia coli* and *Saccharomyces cerevisiae*, and physiological tests have only been reported for uncontrolled shake flask or batch fermentations on rich media or media supplemented with the resveratrol precursors *p*-coumaric acid, phenylalanine or tyrosine (reviewed in [22]). Such cultures, however, do not provide data on strain physiology and kinetics of product formation under industrially relevant process conditions.

The goal of the present study was to investigate the impact of specific growth rate on biomass-specific productivity, product yield, by-product formation and host strain physiology of an *S. cerevisiae* strain that was previously engineered for de novo production of resveratrol from glucose. To this end, (by)product formation, physiology and transcriptome were analysed in steady-state, glucose-limited chemostat cultures grown at different dilution rates.

## Results

### De novo production in an engineered *Saccharomyces cerevisiae* strain: pathway and stoichiometry

**Fig. 1:**
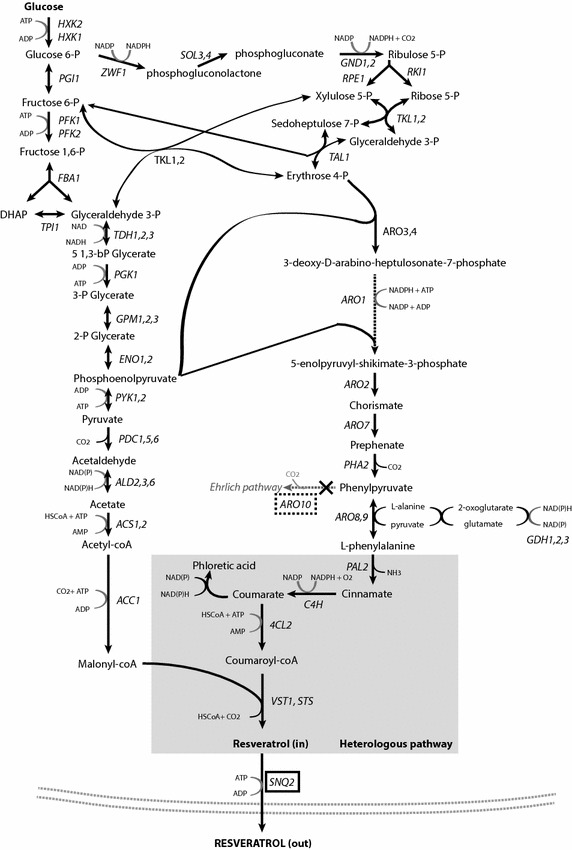
Schematic representation of the engineered *de novo* resveratrol production pathway in an *S. cerevisiae* strain*. Dotted framed boxes* indicate deleted genes and *grey boxes* indicate heterologous genes encoding enzymes in the resveratrol biosynthesis pathway. Phloretic acid is hypothetically formed from coumaric acid via an unidentified reduction reaction [27]

To facilitate interpretation of results from chemostat cultures, a metabolic model covering *S. cerevisiae* central carbon metabolism was expanded to include the resveratrol synthesis pathway present in *S. cerevisiae* strain FS09322 [23]. This strain expresses 5 heterologous plant enzymes that, together, catalyse the conversion of l-phenylalanine and malonyl-CoA to resveratrol (Fig. 1). *PAL2* encodes a phenylalanine ammonia-lyase that converts l-phenylalanine to cinnamate. Subsequently, cinnamate-4-hydroxylyase (encoded by *C4H*) in conjunction with the electron carrier cytochrome b5 (*CYB5*) and a cytochrome p450 reductase (*ATR2*), oxidizes cinnamate to coumarate. A coumarate Co-A-ligase (*4CL2*) covalently binds a Coenzyme-A group to coumarate, forming coumaroyl-CoA. Finally, trihydroxystilbene synthases encoded by *VST1* and *STS* catalyse the reaction of coumaroyl-CoA with three molecules of the precursor malonyl-CoA, thereby forming resveratrol. The *SNQ2* gene, which encodes an ATP-dependent plasma membrane transporter, was overexpressed to optimize resveratrol export. *ARO10*, which encodes a phenylpyruvate decarboxylase was deleted to reduce catabolism of phenylpyruvate via the Ehrlich pathway [24].

Three molecules of malonyl-CoA are required per molecule of resveratrol, which are produced from cytosolic acetyl-CoA. In *S. cerevisiae*, cytosolic acetyl-CoA is formed by the concerted action of glycolysis, pyruvate decarboxylase, acetaldehyde dehydrogenase and acetyl-CoA synthetase. Further, *S. cerevisiae* produces l-phenylalanine via the shikimate pathway from erythrose 4-phosphate and phosphoenolpyruvate. Erythrose-4P formation can occur via the oxidative and the non-oxidative pentose phosphate pathway, depending on the overall pathway balance of redox-cofactor NADPH. Because *S. cerevisiae* has both NADH- and NADPH-dependent acetaldehyde dehydrogenases and glutamate dehydrogenases, 4 different scenarios were incorporated in the stoichiometric model to determine the theoretical maximum yield of resveratrol on glucose (Table 1).

**Table 1:** Maximum theoretical yield of resveratrol on glucose, depending on co-factor specificity of specific enzymes

Active proteins (co-factor specificity)	${\text{Y}}_{\text{P/S}}^{\hbox{max} }$	ATP			
	mol mol^−1^	Glyc (mol)	TCA (mol)	OxPh (mol)	Total (mol)
Ald6 (NADP) and Gdh2 (NAD)	0.284	2.875	0.875	9.25	13
Ald6 (NADP) and Gdh1/3 (NADP)	0.282	2.750	0.750	9.50	13
Ald2/3 (NAD) and Gdh2(NAD)	0.279	2.500	0.500	10.00	13
Ald2/3 (NAD) and Gdh1/3 (NADP)	0.277	2.375	0.375	10.25	13

A stoichiometric model was used to determine the maximum theoretical yield of resveratrol on glucose, and to calculate the ATP demand per mol of product by summing the ATP produced in glycolysis (Glyc), the citric acid cycle (TCA) and by oxidative phosphorylation (OxPh)

In total, 13 mol ATP need to be invested for the production and export of one mole resveratrol, with an estimated in vivo P/O ratio in *S. cerevisiae* of 1.0 [6] and assuming no growth or maintenance requirements. This ATP requirement can be fulfilled by reoxidizing the cytosolic NADH that is formed during resveratrol production by mitochondrial respiration, combined with combustion of up to 0.88 mol of glucose, depending on co-factor specificity of the pathway.

For *S. cerevisiae* grown on glucose, Ald6 has been described as the major acetaldehyde dehydrogenase and Gdh1 as the major glutamate dehydrogenase, which both use NADP as a co-factor [25, 26]. In this case, the pathway yields the overall reaction:

$$3. 5 4 {\text{ Glucose}} + 5. 7 5 {\text{ O}}_{ 2} \to {\text{Resveratrol}} + 7. 2 5 {\text{ CO}}_{ 2} + 1 5. 2 5 {\text{ H}}_{ 2} {\text{O}}.$$

As a result, the maximum theoretical yield of resveratrol on glucose produced in recombinant *S. cerevisiae* equals 0.28 mol mol^−1^.

### Resveratrol production affects yeast physiology

**Fig. 2:**
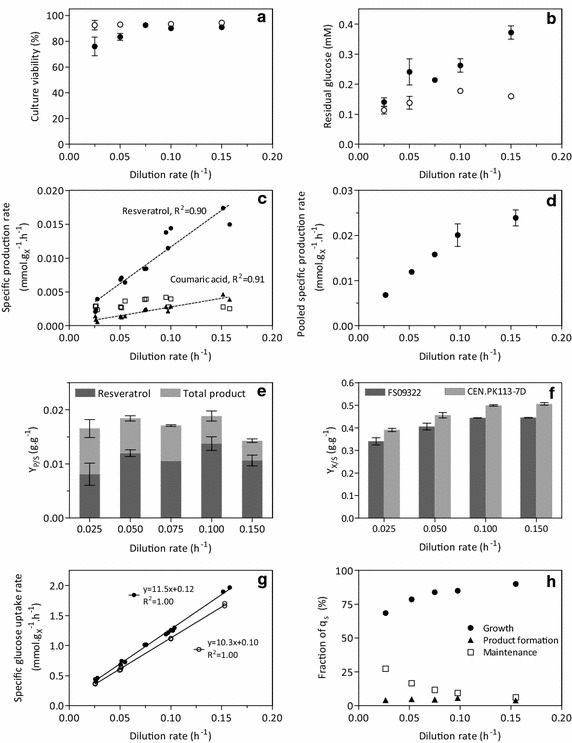
Physiological characteristics of the resveratrol producer FS09322 and of the congenic prototrophic strain CEN.PK113-7D. The data were obtained from aerobic glucose-limited chemostat cultures at various growth rates. **a** Culture viability measured by flow cytometry analysis of PI and CFDA staining (see "Methods" section). *Open symbols* indicate CEN.PK113-7D, *closed symbols* indicate FS09322. **b** Residual glucose concentration, *closed symbols* FS09322, *empty circles* CEN.PK113-7D. **c** Biomass-specific production rate of resveratrol (*circles*) coumaric acid (*triangles*) and phloretic acid (*squares*) in FS09322. **d** Biomass specific production rate of the pooled products (resveratrol + coumaric acid + phloretic acid + cinnamic acid) for FS09322. **e** Resveratrol and total product yield on glucose. **f** Biomass yield on glucose. **g** Biomass specific glucose uptake rate, FS09322 in *closed symbols* and CEN.PK113-7D in *open symbols*. **h** Distribution of the specific substrate uptake (q_s_) in FS09322 as calculated from the Herbert-Pirt equation (Eq. 2) for independent chemostats. In panels **a**–**h**, the shown data represent the average and standard deviation of at least two independent culture replicates for each dilution rate and each strain

Growth and product formation by the resveratrol-producing strain *S. cerevisiae* FS09322 were compared to that of the congenic reference strain CEN.PK113-7D in batch and chemostat cultures. The maximum specific growth rate of strain FS09322, estimated from duplicate shake-flask batch cultures on glucose synthetic medium, was 0.25 h^−1^. This growth rate was 38 % lower than that of the reference strain. In steady-state chemostat cultures grown at a dilution rate of 0.10 h^−1^, not only resveratrol, but also the intermediates coumaric acid, cinnamic acid and phloretic acid were produced by strain FS09322 (see Table 2). In these chemostat cultures, the biomass yield on glucose of strain FS09322 was lower and respiration rates were consistently higher than that of the reference strain. For both strains, viability of these chemostat cultures, as assessed by staining with fluorescent dyes and flow cytometry, was above 90 % (Fig. 2a).

**Table 2:** Physiological characteristics of FS09322 and congenic strain CEN.PK113-7D in aerobic glucose-limited chemostats

	FS09322	CEN.PK113-7D
*Concentrations (µM)*		
Resveratrol	437 ± 39	nd
Coumaric acid	86 ± 11	nd
Phloretic acid	120 ± 20	nd
Cinnamic acid	20 ± 10	nd
*Biomass specific uptake and production rates (mmol g*_*X*_^*−1*^ *h* ^*−1*^)		
Glucose	−1.22 ± 0.03	−1.11 ± 0.01
CO_2_	3.18 ± 0.05	2.65 ± 0.05
O_2_	−3.09 ± 0.03	−2.61 ± 0.02
Pooled products	0.02 ± 0.00	nd
*Yields on glucose*		
Biomass (g g^−1^)	0.44 ± 0.00	0.50 ± 0.00
Resveratrol (mol mol^−1^)	0.011 ± 0.001	
Pooled products (mol mol^−1^)	0.016 ± 0.002	

A dilution rate of 0.10 h^−1^ was applied. Data represent the average ± standard deviation of measurements on three independent chemostats for resveratrol producing strain FS09322 and two independent chemostats for congenic reference strain CEN.PK113-7D

*nd* Not detected

The formation rates of the by-products coumaric acid, phloretic acid and cinnamic acid were relatively low (Fig. 2c). Still, it was conceivable that their formation contributed to the reduced biomass yield of strain FS09322 in the chemostat cultures, e.g. via weak-acid uncoupling. To investigate this possibility, glucose-limited chemostat cultures of the reference strain CEN.PK113-7D were supplemented with the products of the resveratrol pathway at concentrations close to their solubility in water. None of these compounds were consumed and they affected neither the biomass yield on glucose nor the culture viability (above 90 % in all cultures, Table 3).

**Table 3:** Impact of resveratrol pathway products on physiology of CEN.PK113-7D

	Concentration (mg l^−1^)	Biomass yield (g g^−1^)	q_s_ (g gX^−1^ h^−1^)	qCO_2_ (g gX^−1^ h^−1^)	Viability (%)
Reference	–	0.49 ± 0.00	−1.13 ± 0.00	2.62 ± 0.01	92 ± 1
Phloretic acid	253 ± 1	0.50 ± 0.00	−1.12 ± 0.01	2.58 ± 0.06	91 ± 3
Cinnamic acid^a^	154 ± 18	0.47 ± 0.00	−1.18 ± 0.02	–	94 ± 1
Coumaric acid	91 ± 5	0.49 ± 0.00	−1.14 ± 0.00	2.67 ± 0.00	93 ± 1
Resveratrol	6.3 ± 0.8	0.49 ± 0.00	−1.15 ± 0.00	2.68 ± 0.02	95 ± 0

The prototrophic reference strain CEN.PK113-7D was grown in aerobic, glucose-limited chemostat cultures in the absence or presence of phloretic acid, cinnamic acid, coumaric acid or resveratrol. Data represent the average ± standard deviation of measurements on two independent chemostat cultures. Phloretic acid, cinnamic acid, coumaric acid or resveratrol were not consumed by CEN.PK113-7D in chemostat cultures

^a^Repeated efforts to obtain a steady state with cultures grown in the presence of cinnamic acid consistently resulted in periodic variations in the oxygen uptake and carbon dioxide production

### Specific growth rate affects product formation

The effect of specific growth rate on resveratrol production by *S. cerevisiae* was analyzed in steady-state glucose-limited chemostat cultures of the resveratrol-producing strain FS09322 and the reference strain CEN.PK113-7D. Independent replicate cultures of each strain were grown at 0.025 h^−1^, 0.05 h^−1^, 0.075 h^−1^ (FS09322 only), 0.10 h^−1^ and 0.15 h^−1^. At these dilution rates, sugar dissimilation in the chemostat cultures was completely respiratory, as evident from the absence of ethanol in culture supernatants and a respiratory quotient (q_CO2_/q_O2_) that was close to unity. Culture viability remained above 90 % for both strains at dilution rates above 0.075 h^−1^. However, below this dilution rate, viability of strain FS09322 decreased, reaching a value of ca. 76 % at a dilution rate of 0.025 h^−1^ (Fig. 2a). This implied that, especially at low dilution rates, the specific growth rate no longer exactly matched the dilution rate. For the sake of clarity, we will refer to the value of the dilution rate throughout this paper. Residual glucose concentrations in culture supernatants remarkably differed between the two strains. While the residual glucose concentration in cultures of the reference strain remained between 0.1 and 0.17 mM over this range of dilution rates, it strongly increased with increasing dilution rate in cultures of the resveratrol producer, reaching 0.37 ± 0.02 mM at the highest dilution rate tested (Fig. 2b).

The biomass-specific resveratrol production rate exhibited a strong positive correlation with the specific growth rate in strain FS09322 (Fig. 2c, linear regression R^2^ > 0.9). A similar positive correlation was found for the specific coumaric acid production rate (Fig. 2c) and for the pooled phenylpropanoid-pathway-derived products (resveratrol, coumaric acid, cinnamic acid and phloretic acid, Fig. 2d). This biomass-specific pooled product formation rate reached 0.024 ± 0.002 mmol (g biomass)^−1^ h^−1^ at the highest tested dilution rate (0.15 h^−1^). Conversely, the biomass-specific production of phloretic acid, presumably formed from coumaric acid via an unidentified reduction reaction [27], was not correlated to the specific growth rate. The yield of total products on glucose was stable around 0.018 g g^−1^ at dilution rates ranging from 0.025 to 0.10 h^−1^, but decreased to 0.014 ± 0.001 g g^−1^ at a dilution rate of 0.15 h^−1^ (Fig. 2e). The maximum resveratrol yield was obtained at a dilution rate of 0.10 h^−1^ and equaled 0.011 ± 0.001 mol mol^−1^ (Table 2), representing 4.1 % of the maximum theoretical yield of 0.28 mol mol^−1^ (see above).

The difference in biomass yield between the resveratrol-producing strain FS09322 and the reference strain CEN.PK113-7D that was observed at a dilution rate of 0.10 h^−1^ (Table 2) was also found at the other dilution rates (Fig. 2f). The average difference in biomass yield between the two strains was 12 %, while q_CO2_ and q_O2_ increased on average by 21 and 22 %, respectively (Additional file 1: Figure S1). These differences were significant (*p* value < 0.05) for all dilution rates above 0.025 h^−1^.

### Expression of the resveratrol production pathway does not impact cellular maintenance energy requirements

Growth-rate-independent maintenance energy requirements (m_s_) of the resveratrol producing strain FS09322 and the reference strain CEN.PK113-7D were estimated by plotting biomass-specific glucose consumption rates as a function of specific growth rate [5, 28]. This yielded similar values for m_s_ of 0.12 ± 0.02 mmol (g biomass)^−1^ h^−1^ for strain FS09322 and 0.10 ± 0.01 mmol (g biomass)^−1^ h^−1^ for strain CEN.PK113-7D (Fig. 2g). When assuming a P/O ratio of 1.0 [6] in fully respiratory metabolism, the maintenance energy requirements can be translated to values of 1.92 ± 0.32 and 1.52 ± 0.15 mmol g^−1^ h^−1^ ATP for FS09322 and CEN.PK113-7D, respectively.

The Herbert-Pirt equation [5] specifies that, in energy-source-limited chemostat cultures, the biomass-specific substrate uptake rate (q_s_) is distributed over growth, expressed as $\left( {\frac{\upmu }{{{\text{Y}}_{\text{X/S}}^{ \hbox{max} } }}} \right)$, maintenance (m_s_) and product formation, expressed as $\sum\limits_{\text{i}} {\left( {\frac{{{\text{q}}_{{{\text{p}}_{\text{i}} }} }}{{{\text{Y}}_{{{\text{P}}_{\text{i}} / {\text{S}}}}^{ \hbox{max} } }}} \right)}$, which is the sum of all anabolic products excreted by the organism. The reference strain CEN.PK113-7D invests all glucose in growth and maintenance and does not make product, which simplifies the Herbert-Pirt relation to Eq. 1:

1 $${\text{q}}_{\text{s}} = \frac{\upmu }{{{\text{Y}}_{\text{X/S}}^{ \hbox{max} } }} + {\text{m}}_{\text{s}} .$$

Because strain FS09322 also invests part of the consumed glucose in product formation and excretion, the production term has to be added in the equation, resulting in Eq. 2:

2 $${\text{q}}_{\text{s}} = \frac{\upmu }{{{\text{Y}}_{\text{X/S}}^{ \hbox{max} } }} + {\text{m}}_{\text{s}} + \sum\limits_{\text{i}} {\left( {\frac{{{\text{q}}_{{{\text{p}}_{\text{i}} }} }}{{{\text{Y}}_{{{\text{P}}_{\text{i}} / {\text{S}}}}^{ \hbox{max} } }}} \right)} .$$

For both the reference strain and the producing strain, the substrate uptake rate (q_s_) was experimentally determined at each dilution rate (Fig. 2g). Furthermore, the substrate requirements for maintenance purposes (m_s_) were estimated for both strains as described above. For the production strain, the maximum theoretical product yield was calculated using the stoichiometric model, and the specific production rates were determined experimentally for all products (Fig. 2c). Subsequently, Eq. 2 was used to calculate the substrate fractions distributed between product formation (q_s_ divided by the production term), maintenance energy requirements (q_s_ divided by m_s_), and growth (remaining fraction), for strain FS09322 at each tested dilution rate (Fig. 2h). Accordingly, in the resveratrol producer the fraction of substrate invested in maintenance processes increased at low growth rates, reaching 27 ± 2 % of the total specific substrate consumption at the lowest dilution rate. Conversely, the fraction of the glucose channeled towards (pooled) product formation was remarkably growth-rate independent at 4.5 ± 0.5 % (Fig. 2h).

### Specific growth rate differentially affects gene expression in a resveratrol producer and a reference strain

**Fig. 3:**
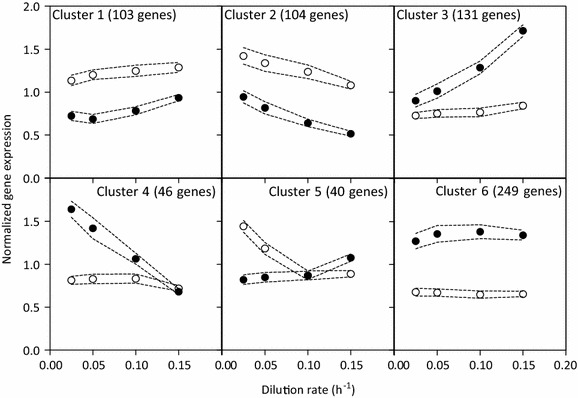
K-mean clustering of the 673 genes with differential expression profiles between FS09322 and CEN.PK113-7D. The data results from a dilution range of independent chemostat cultures (q-value for differential expression profiles below 0.005, see "Methods" section). For each cluster, the averaged normalized expression values are depicted for the resveratrol producing *S. cerevisiae* FS09322 (*black circles*) and for its congenic reference strain CEN.PK113-7D (*open symbols*) for the different dilution rates. The *grey dotted lines* exhibit the average standard error of these values

To assess the impact of expressing a resveratrol pathway on the transcriptome of *S. cerevisiae*, genome-wide transcript levels of the resveratrol producer and the reference strain were compared over the whole range of dilution rates. Growth rate is known to strongly affect gene expression [29]. As expected, in both strains this typical transcriptome response was observed with an overrepresentation of genes involved in biosynthetic processes and protein synthesis among the genes which expression was negatively correlated to growth rate, and an enrichment for stress-responsive genes among the genes which expression was positively correlated to growth rate. More interesting was the set of genes that were specifically differentially expressed in the resveratrol producer as compared to the reference strain. 673 genes with significantly divergent expression profiles (q-value <0.005, see "Methods" section) in the resveratrol-producing and reference strain were identified and classified in 6 clusters according to their expression profile (Fig. 3). Only gene expression profiles in clusters 1 and 6 showed no obvious correlation with dilution rate, but revealed a strong, consistent difference in expression between the two strains.

Remarkably, a strong overrepresentation of genes whose transcript levels were previously identified as being glucose-responsive were found in cluster 2 (34 out of 104 genes, p-value of 5.7E−11), cluster 3 (44 out of 131 genes, p-value of 5.5E−14) and cluster 6 (44 out of 249 genes, p-value 1.8E−4) (Table 4). Genes known to be down-regulated in response to high glucose levels were overall more strongly down-regulated in the resveratrol producing strain with increasing growth rate (Cluster 2).

**Table 4:** Overrepresentation of MIPS categories among the clusters of differentially expressed genes (see Fig. 3)

Cluster	Functional catagory	Number of genes in cluster	Total number of genes in category	Bonferroni-corrected p-value^a^
1	Ribosome biogenesis	18	343	1.68E−2
2	Glucose responsive DOWN	34	565	5.73E−11
	Lipid, fatty acid and isoprenoid metabolism	20	291	7.10E−05
	ENERGY	18	360	3.77E−02
3	Glucose responsive UP [58]	44	589	5.48E−14
4	No significant terms			
5	No significant terms			
6	Glucose responsive UP [58]	44	589	1.78E−4

^a^A statistical Bonferroni-corrected p-value threshold for overrepresentation of 0.05 was applied

Several structural genes that encode enzymes involved in de novo production of resveratrol and its precursors from glucose were differentially expressed in the resveratrol-producing and reference strain. *PDC1*, which is involved in cytosolic acetyl-CoA synthesis and, thereby, in malonyl-CoA supply (cluster 3), as well as *ARO7* and *ARO9* (clusters 3 and 6 respectively), which are involved in phenylalanine biosynthesis, displayed higher expression levels in strain FS09322 than in the reference strain. *TKL1* (cluster 3) and *RKI1* (cluster 1) encoding a transketolase and ribose-5-phosphate ketol-isomerase respectively, two key enzymes in the pentose phosphate pathway, were also differentially expressed in the two strains. *SNQ2* (multi-drug transporter) and *CYB5* (cytochrome b5), of which additional copies were integrated in the genome of the resveratrol producing strain, were unexpectedly not significantly differentially expressed. *PDR12*, which encodes for another multidrug ABC transporter displayed higher expression levels in the resveratrol production strain than in the reference strain (cluster 3). Furthermore, cluster 1 was enriched for genes encoding ribosomal proteins (18 out of 343 genes, p-value of 1.7E−2), suggesting a constitutively lower expression of these genes in FS09322 as compared to CEN.PK113-7D. However, measurement of whole cell protein content did not show differences between the two strains (Additional file 2: Figure S2). Finally, gene expression levels indicated that *ALD6* (100-fold higher expression than *ALD2* and *ALD3*) and *GDH1* (6-fold higher expression than *GDH2* and *GDH3*) encoded the main acetaldehyde and glutamate dehydrogenases respectively in our cultivation conditions, as hypothesized earlier.

## Discussion

### Resveratrol yield in chemostat cultures

Earlier studies on resveratrol production by yeast did not allow for a quantitative analysis of product yields on glucose, as the strains used lacked a complete biosynthetic pathway and were fed with coumaric acid or aromatic amino acids as precursors [22]. The present study describes a first quantitative analysis of an *S. cerevisiae* strain that was engineered for de novo production of resveratrol from glucose. In glucose-limited, aerobic chemostat cultures of *S. cerevisiae* FS09322, the resveratrol yield on glucose was approximately 0.011 ± 0.002 g g^−1^ (Fig. 2e), irrespective of the specific growth rate. The resveratrol yield on glucose found in this study is ca. three-fold higher than the product yield in batch cultures of an *S. cerevisiae* strain engineered for production of naringenin, a product that is also derived from the phenylpropanoid pathway [30]. However, the experimental resveratrol yield is only ca. 4 % of the maximum theoretical yield of 0.28 mol mol^−1^, indicating that there is substantial room for further improvement of resveratrol yields. One aspect that should be addressed in this context is formation of by-products derived from the phenylpropanoid pathway. Excretion of coumaric acid and phloretic acid by the resveratrol-producing strain (Fig. 2c) represents a loss of approximately one third of the carbon entering the phenylpropanoid pathway. These by-products were also found in cultures of an *S. cerevisiae* strain engineered for naringenin production [30], indicating that their formation is a generic challenge in engineering of the phenylpropanoid pathway. Addressing this carbon loss by further metabolic engineering is complicated by the fact that the enzyme(s) responsible for phloretic acid synthesis in *S. cerevisiae*, possibly through a NAD(P)H-dependent reduction of coumaric acid, is (are) as yet unknown [27, 30]. Other metabolic engineering strategies that may contribute to improved resveratrol production include deregulation of aromatic amino acid metabolism [31], engineering flux and energy coupling of cytosolic acetyl-CoA synthesis [32, 33], and expression of a deregulated allele of *ACC1* [34].

### Resveratrol productivity is growth-rate dependent

The relationship between specific growth rate (μ) and biomass-specific productivity (q_p_) is a key parameter in the design of aerobic fed-batch processes for microbial product formation. We observed a strong positive correlation between q_p_ and μ in aerobic, glucose-limited cultures of an engineered, resveratrol-producing strain of *S. cerevisiae.* Well documented q_p_-µ relationships for engineered yeast strains are scarce. Similar positive correlations between q_p_ and μ relations as identified in this study were found for heterologous production of proteins by engineered yeasts [15, 35] and for production of ethylene by an *S. cerevisiae* strain expressing a heterologous ethylene-forming enzyme [15]. Measurements at two dilution rates in aerobic, glucose-limited chemostat cultures of an *S. cerevisiae* strain engineered for production of α-santalene, a product derived from the isoprenoid pathway, also indicated a positive correlation of these parameters [36]. These processes share an ATP requirement for product formation, as well as the use of precursors that also play a key role in biomass synthesis (in the case of resveratrol production, phenylalanine and malonyl-CoA). The same mechanisms that tune down anabolic routes as the growth rate decreases most probably also tune down product formation. In glucose-limited cultures of *S. cerevisiae*, the strong correlation of specific growth rate with the intracellular concentrations of key metabolic intermediates [37], provides a plausible explanation for the observed positive correlation of q_p_ and μ. In view of the central role of many of the involved precursors in central metabolism, breaking this correlation represents a major challenge for metabolic engineers and synthetic biologists [28]. Conversely to q_p_, the fraction of substrate invested in product formation is rather insensitive to growth rate. It is remarkable that, while yeast cells have to carefully allocate their limited carbon and energy resources between biomass formation and maintenance, the fraction of resources channelled towards product formation remains unchanged over the tested growth rate range.

### High maintenance-energy requirements are caused by process conditions rather than by resveratrol production

Large-scale aerobic fed-batch processes invariably involve a decreasing specific growth rate. Maintenance-energy requirements (m_s_, mmol glucose (g biomass)^−1^ h^−1^) can therefore have a strong impact on the performance of microbial strains in such processes. This was also observed in chemostat cultures of the resveratrol-producing strain. At a dilution rate of 0.025 h^−1^ which, with a culture viability of 76 %, corresponded to a specific growth rate of ca. 0.03 h^−1^, 27 % of the glucose fed to the cultures was respired to meet cellular maintenance energy requirement, rather than channelled towards growth or resveratrol production (Fig. 2h). Reducing this loss of substrate carbon, for example by choice of a microbial host with a lower maintenance-energy requirements, can have a significant impact on product yields in industrial fed-batch processes.

When analysed under the conditions employed in this study, m_s_ values for a resveratrol-producing strain and a congenic reference strain were not significantly different. Moreover, control experiments confirmed that products originating from the phenylpropanoid pathway that were excreted by the resveratrol-producing strain did not affect biomass yields of the reference strain at pH 6.5 (Table 3). Although formation of by-products should ultimately be prevented by further engineering, our data indicate that *S. cerevisiae* is remarkably tolerant towards these by-products. Coumaric acid, cinnamic acid and phloretic acid have previously been reported to suppress bacterial growth (e.g. *Lactobacillus plantarum* at pH 6.5, [27]). Tolerance of *S. cerevisiae* is, however, likely to be strongly pH dependent. At a pH of 4.0, growth of a wine strain of *S. cerevisiae* was strongly inhibited by 35 mg l^−1^ cinnamic acid [38], suggesting that cinnamic acid induces toxicity by diffusion of the undissociated form across the yeast membrane, as has been described for benzoic acid and other weak acids [39].

Although the m_s_ values estimated for the resveratrol-producing strain and the reference strain were not significantly different (Fig. 2g), they were 40–50 % higher than found in earlier studies with *S. cerevisiae*. An ATP requirement for maintenance (m_ATP_) of 1.5 ± 0.15 mmol g biomass^−1^ h^−1^ ATP was estimated for *S. cerevisiae* strain CEN.PK113-7D in this work. Rogers and Stewart [40] estimated an m_ATP_ of 1.12 mmol g biomass^−1^ h^−1^ ATP from aerobic, glucose-limited chemostat cultures of a diploid wild-type *S. cerevisiae* strain. Using anaerobic chemostat and retentostat cultures of *S. cerevisiae* CEN.PK113-7D, Boender et al. [28] calculated an m_ATP_ of 1.0 mmol ATP g biomass^−1^ h^−1^ for this strain. The higher maintenance energy requirement observed in our experiments may be related to the elevated concentrations of copper in the medium, which were needed to induce the *PAL2* gene in the resveratrol-producing strain. Because copper is toxic at higher concentrations [41], the use of copper-dependent induction systems should preferably be avoided in bioprocesses.

### Resveratrol production pathway impacts expression levels of upstream genes

Among the genes encoding enzymes directly involved in phenylalanine biosynthesis, *TKL1, ARO7*, and *ARO9* displayed significantly higher expression levels and *RKI1* lower expression levels in the resveratrol producing strain than in the reference strain. These transcriptional differences may result from the genetic engineering performed to channel carbon towards resveratrol formation. Resveratrol production via the oxidative branch of the pentose phosphate pathway (in which *RKI1* encodes an intermediate step) results in net NADPH production (see stoichiometry). Transketolase, encoded by *TKL1*, offers a non-oxidative pathway for pentose phosphate production from glycolytic intermediates (Fig. 1). The antagonistic regulation of *TKL1* and *RKI1* may therefore respond to a need for redox balancing in the resveratrol producer. Closer to phenylalanine, expression of *ARO9* is activated by aromatic amino acids and expression of *ARO7* is repressed by tyrosine [42]. Increased expression of these two genes in the resveratrol producer may thus reflect alterations in intracellular amino acid concentrations. In addition, transcript levels of the multidrug transporter Pdr12 were consistently higher in the resveratrol producing strain than in the reference strain irrespective of growth rate. Expression of *PDR12* is induced by weak organic acids, which suggests that intermediates of the resveratrol pathway (coumaric acid, cinnamic acid and/or phloretic acid) may induce *PDR12* [43]. Even though resveratrol production levels were relatively low, genetic engineering and heterologous resveratrol production had therefore an impact on expression of key endogenous enzymes involved in the de novo pathway.

### Differences in relative growth rate result in a glucose-dependent transcriptome response

Both the resveratrol producing strain and the congenic reference strain showed a positive correlation between specific growth rate and expression of genes involved in anabolism, a relationship that has been identified before [29]. Furthermore, a negative correlation was observed for genes involved in reaction to stress, a response known to decrease with growth rate [29].

Comparison of the two strains, however, showed that the most prominent differences in gene expression involved a set of genes known to respond to extracellular glucose concentration. This response agreed with the residual glucose concentration, which showed a pronounced correlation with specific growth rate in cultures of the resveratrol producer (Fig. 2b). In steady-state glucose-limited chemostat cultures, the residual glucose concentration (C_S_) is dependent on the specific growth rate (µ) (which in steady-state chemostats equals the dilution rate), the maximum specific growth rate (µ_max_) under the experimental conditions, and the microorganism’s substrate saturation constant for glucose (K_s_), according to kinetics first proposed by Monod [44].

$$\upmu = \upmu_{\hbox{max} } \frac{{{\text{C}}_{\text{s}} }}{{{\text{K}}_{\text{s}} {\text{ + C}}_{\text{s}} }}$$

The maximum specific growth rate of the resveratrol producer was 38 % lower than that of the reference strain. At each growth rate tested in chemostat, this strain therefore operated closer to its µ_max_ than the reference strain. The resulting higher relative specific growth rate (µ/µ_max_) is consistent with the higher residual glucose concentrations in cultures of the resveratrol producing strain [45]. While chemostat cultivation is a powerful and widely used tool to compare strains with different µ_max_ at the same specific growth rate, the potential impact of differences in relative growth rate has hitherto been largely overlooked. In a recent study, Hebly and co-workers, exposing *S. cerevisiae* to temperature oscillations in glucose-limited continuous cultures, observed that the relative growth rate of yeast at different temperatures had a stronger impact on physiology and transcriptome than temperature itself [45]. The present study provides a clear illustration of the importance of considering relative as well as absolute growth rates in chemostat-based comparisons of different microbial strains.

## Conclusions

Low specific growth rates are a common constraint in industrial fed-batch processes for the microbial production of compounds whose formation from glucose requires a net input of ATP. Glucose-limited chemostat cultivation of a recombinant resveratrol-producing *S. cerevisiae* strain demonstrated a strong correlation between recombinant resveratrol production from glucose and specific growth rate. By-product formation was identified as a clear priority for future research on improving resveratrol yields. Furthermore, this study underlined the impact of specific growth rate on the distribution of glucose, the carbon and energy source, over growth, maintenance requirements and product formation. The results emphasize the importance of metabolic engineering strategies that enable uncoupling of product formation and growth in the microbial production of ATP-requiring compounds and of minimizing maintenance energy requirements in such processes.

## Methods

### Strains

The prototrophic resveratrol-producing strain *Saccharomyces cerevisiae* FS09322 [46], was obtained from Fluxome Sciences, Stenløse, Denmark. Requests for academic use of strain FS09322 under a Materials Transfer Agreement should be addressed to Evolva (Reinach, Switzerland). The congenic prototrophic strain CEN.PK113-7D (*MATa, MAL2*-*8c, SUC2*) was used as a reference [47]. Stock cultures of *S. cerevisiae* CEN.PK113-7D were grown in 500 ml shake flasks on 100 ml YPD medium (10 g l^−1^ Bacto yeast extract, 20 g l^−1^ Bacto peptone and 20 g l^−1^d-glucose). After addition of glycerol (20 % v/v) to early stationary phase cultures, 2 mL aliquots were stored at −80 °C. Stock cultures of *S. cerevisiae* FS09322 were grown in 500 ml shake flasks on 100 ml synthetic medium [48] set to pH 6.0 with 2 M KOH, and containing 20 g l^−1^d-glucose. 2 mL aliquots were stored at −80 °C.

### Media and cultivation methods

Shake-flask cultures were grown in an orbital shaker at 200 rpm and at 30 °C in synthetic medium [48], set to pH 6.0 with 2 M KOH prior to sterilization and supplemented with 20 g l^−1^d-glucose. Pre-cultures were grown in 500 ml shake flasks containing 100 ml of the same medium, inoculated with a 2-ml glycerol stock. Aerobic chemostat cultivation was performed in 2 litre bioreactors (Applikon, Delft, the Netherlands) equipped with a level sensor to maintain a constant working volume of 1 litre. The culture temperature was controlled at 30 °C and dilution rates between 0.025 h^−1^ and 0.15 h^−1^ were set by controlling the flow rate. Chemostat cultures of both CEN.PK113-7D and FS09322 were grown on synthetic medium [48], supplemented with 7.5 g l^−1^d-glucose, 0.3 g l^−1^ Struktol J673 antifoam (Schill and Scheilacher AG, Hamburg, Germany), and 0.015 g l^−1^ copper sulfate pentahydrate (copper concentrations in the medium required for induction of *CUP1p* controlled *PAL2* were optimized for specific resveratrol production rate in batch to a concentration of 0.015 g l^−1^, without affecting the µ_max_ of FS09322). The pH was kept constant at 6.5 by automatic addition of 2 M KOH. Cultures were sparged with air (0.5 l min^−1^) and stirred at 800 rpm. Chemostat cultures were assumed to be in steady state when, after at least 6 volume changes, the culture dry weight and specific carbon-dioxide production rate changed by less than 3 % over 2 consecutive volume changes. Steady-state samples were taken between 10 and 16 volume changes after inoculation to minimize the impact of evolutionary adaptation. Carbon recoveries for independent chemostats were >95 %. For the growth rate range study, 15 independent chemostats were performed with FS09322, three at a dilution rate of 0.025 h^−1^, three at 0.05 h^−1^, two at 0.075 h^−1^, five at 0.10 h^−1^, and two at 0.15 h^−1^. For CEN.PK113-7D, ten independent chemostats were performed, two at 0.025 h^−1^, four at 0.05 h^−1^, two at 0.10 h^−1^ and two at 0.15 h^−1^. For the study on the effect of (by-)products, reference strain CEN.PK113-7D was grown in independent duplicate glucose-limited chemostats performed at a dilution rate of 0.10 h^−1^ in synthetic medium [48] supplemented with either resveratrol (6.3 ± 0.8 mM), coumaric acid (91 ± 5 mM), phloretic acid (253 ± 1 mM) or cinnamic acid (154 ± 18 mM).

### Determination of substrate, metabolites and biomass concentration

Culture dry weight was measured by filtering 10 mL of culture broth over pre-dried and pre-weighed membrane filters (pore size 0.45 um, Gelman Science), which were then washed with demineralized water, dried in a microwave oven (20 min, 350 W) and weighed again. Supernatants were obtained by centrifugation of culture samples (3 min at 20.000 g) and analysed by high-performance liquid chromatograph (HPLC) analysis on a Waters Alliance 2690 HPLC (Waters, Milford, MA) equipped with a Bio-Rad HPX 87H ion exchange column (BioRad, Veenendaal, The Netherlands), operated at 60 °C with 5 mM H_2_SO_4_ as the mobile phase at a flow rate of 0.6 ml min^−1^. Detection was by means of a dual-wavelength absorbance detector (Waters 2487) and a refractive index detector (Waters 2410). For measurement of phenylpropanoic compounds, culture samples were diluted with an equal volume of 50 % ethanol. After vigorous mixing, cells were spun down at 20.000 g for 3 min. The supernatant was analysed on a Waters 2695 separation module and a Waters 996 photodiode array detector. Resveratrol, phloretic acid, coumaric acid, phenylethanol, and cinnamic acid were measured at 306, 275, 309, 214 and 277 nm, respectively, using an Agilent Zorbax SB-C18 Column (4.6 × 5.0, 3.5 micron) operated at 30 °C. A gradient of acetonitrile and 20 mM KH_2_PO_4_ (pH 2) with 1 % acetonitrile was used as eluent, at a flow rate of 1 ml·min^−1^, increasing from 0 to 10 % acetonitrile in 6 min followed by an increase to 40 % acetonitrile until 23 min. From 23 min to 27 min, 100 % KH_2_PO_4_ was used as eluent. Resveratrol, coumaric acid, cinnamic acid, phloretic acid and phenylethanol standards for calibration were obtained from Sigma Aldrich (Sigma-Aldrich, Zwijndrecht, The Netherlands). Residual glucose concentrations in glucose-limited chemostat cultures were analysed after rapid quenching with cold steel beads [49], using an enzymatic glucose kit (Roche, Almere, The Netherlands, no. 0716251).

### Gas analysis

The exhaust gas from chemostat cultures was cooled with a condenser (2 °C) and dried with a PermaPure Dryer (model MD 110-8P-4; Inacom Instruments, Veenendaal, the Netherlands) prior to online analysis of carbon dioxide and oxygen with a Rosemount NGA 2000 Analyser (Baar, Switzerland). Exhaust gas flow rates, biomass-specific carbon dioxide production rates and oxygen consumption rates were calculated as described previously [50].

### Viability assays

Chemostat cultures were assayed for viability using the *Funga*Light AM-CFDA (acetoxymethyl ester 5-carboxyfluorescein diacetate)/propidum iodide yeast viability kit (Invitrogen, Carlsbrad, CA) by counting 10,000 cells on a Cell Lab Quanta SC MPL flow cytometer (Beckman Coulter, Woerden, Netherlands) as described previously [51]. AM-CFDA is a cell-permeant substrate for an intracellular non-specific esterase activity. Hydrolytic cleavage of the lipophilic blocking and diacetate groups of AM-CFDA results in a green fluorescence in metabolically active cells. Propidium Iodide intercalates with DNA in cells with a compromised cell membrane, which results in red fluorescence.

### Protein determination

A fresh sample of the culture containing 50 mg biomass was centrifuged, and the pellet was washed twice with distilled water and resuspended in 5 ml of water. The concentrate was boiled in 1 M NaOH (final concentration) for 10 min and subsequently cooled on ice. Samples were 10 times diluted in distilled water and further processed according to the protocol for Bradford Quick Start Protein Assay (Bio-Rad, Veenendaal, Netherlands). Absorbance of samples was measured at 595 nm. Dried bovine serum albumin (Sigma-Aldrich, Zwijndrecht, The Netherlands) was used as a standard.

### Transcriptome analysis

Microarray analysis was performed with samples from independent duplicate steady-state chemostat cultures of *S. cerevisiae* strains FS09322 and CEN.PK113-7D grown at four different dilution rates, comprising a total dataset of 16 microarrays. Sampling from chemostat cultures for transcriptome analysis was carried out by using liquid nitrogen for rapid quenching of mRNA turnover [52]. Prior to RNA extraction, samples were stored in a mixture of phenol/chloroform and TEA buffer at −80 °C. Total RNA extraction, isolation of mRNA, cDNA synthesis, cRNA synthesis, labelling and array hybridization was performed as described previously [53], with the following modifications. To chelate the copper present at 4 mg/L in the culture medium and thereby prevent copper-induced mRNA degradation [54], EDTA was added to defrosting samples at a final concentration of 80 mM. The quality of total RNA, cDNA, aRNA and fragmented aRNA was checked using an Agilent Bioanalyzer 2100 (Agilent Technologies, Santa Clara, CA). Hybridization of labelled fragmented aRNA to the microarrays and staining, washing and scanning of the microarrays was performed according to Affymetrix instructions (EukGE_WS2v5).

The 6383 yeast open reading frames were extracted from the 9335 transcript features on the YG-S98 microarrays. All microarray data used in this study are available via GEO series accession number GSE65942. To allow comparison, all expression data were normalized to a target value of 240 using the average signal from all gene features. To eliminate variation in genes that are not expressed, genes with expression values below 12 were set to 12 and the gene features for which the maximum expression was below 20 for all 19 arrays were discarded. The average deviation of the mean transcript data of replicate chemostats was approximately 14 %, similar to the reproducibility usually observed in replicate steady state chemostat cultures [23]. The expression of housekeeping genes *ACT1, HHT2, SHR3, PDA1* and *TFC1* [55] remained stable for both strains at all tested growth rates (average coefficient of variation 12 ± 2 % see Additional file 3: Figure S3).

EDGE version 1.1.291 [56] was used to perform a differential expression analysis based on gene expression profiles across the different dilution rates, using strains and dilution rates as covariates. Expression profiles with a false discovery rate below 0.005 (p-value 0.0025) were considered as significantly differently expressed between the two strains and were clustered with k-means clustering using positive correlation as distance metric (Expressionist Pro version 3.1, Genedata, Basel, Switzerland).

Gene expression clusters were analysed for overrepresentation of functional annotation categories from the Munich Information Centre for Protein Sequences (MIPS) database (http://www.mips.gsf.de/genre/proj/yeast), based on the hypergeometric distribution analysis tool described by Knijnenburg et al. [57]. Additional categories were searched for enrichments, that consist of a set of 589 genes transcriptionally up-regulated (designated Glucose responsive UP) and 565 genes transcriptionally down-regulated (designated Glucose responsive DOWN) upon addition of excess glucose to glucose-limited chemostat cultures of *S. cerevisiae* (aerobic cultures, same experimental set-up and strain background as in the present study) [58].

### Stoichiometric calculations

The maximum yield of resveratrol on glucose was calculated using a compartmented stoichiometric model for aerobic growth of *S. cerevisiae* on glucose [23]. The model was extended to allow resveratrol production by incorporating the reactions catalyzed by: l-phenylalanine ammonia lyase, cinnamate 4-hydroxylyase, coumarate CoA ligase, reservatrol synthase and the ATP-binding cassette transporter Snq2 for export of resveratrol from the cells. The list of additional reactions can be found in Additional file 4. The resulting model did not contain parallel reactions, and when the growth rate was set to zero the only degree of freedom was the rate of resveratrol production.

By setting the growth rate to zero and the resveratrol production to a certain fixed value the flux distribution and the net requirement of glucose and oxygen were calculated for different network options, that is NADPH production via Ald6 or the pentose phosphate pathway, combined with different cofactor specificities of glutamate dehydrogenase (NADH or NADPH). From these, the maximum yields of resveratrol on glucose, and the ATP requirement for resveratrol biosynthesis were calculated. For all calculations the P/O ratio for respiratory ATP production was set to 1.0.

## Additional files

Additional file 1:
**Figure S1.** Specific CO_2_ production and specific O_2_ uptake rates of the resveratrol producing *S. cerevisiae* strain FS09322 and the isogenic strain CEN.PK113-7D. Closed symbols indicate the resveratrol producing *S. cerevisiae* strain FS09322. Open symbols indicate isogenic strain CEN.PK113-7D. Each data point represents results from an individual chemostat. Additional file 2:
**Figure S2.** Protein content of the resveratrol producing *S. cerevisiae* strain FS09322 and its isogenic strain CEN.PK113-7D. Open symbols indicate strain CEN.PK113-7D, close symbols indicate strain FS09322. The shown data represent the average and standard deviation of two independent culture replicates for each dilution rate and each strain. Additional file 3:
**Figure S3.** Averaged normalized gene expression of housekeeping genes^1^ for *S. cerevisiae* strain FS09322 and CEN.PK113-7D. Dotted bars indicate 20 % variation around normalized expression. ^1^Teste MA, Duquenne M, Francois JM, Parrou JL: Validation of reference genes for quantitative expression analysis by real-time RT-PCR in *Saccharomyces cerevisiae*. *BMC Mol Biol* 2009, 10:99. Additional file 4: Additional reactions for incorporation of resveratrol biosynthesis in the stoichiometric model.

## Abbreviations

µ: specific growth rate
µ_max_: maximum specific growth rate
q_p_: specific production rate
q_s_: specific substrate consumption rate
${\text{Y}}_{\text{X/S}}^{ \hbox{max} }$: maximum biomass yield on substrate
${\text{Y}}_{{{\text{P}}_{i} / {\text{S}}}}^{ \hbox{max} }$: maximum product yield on substrate
m_s_: substrate requirements for maintenance
m_ATP_: ATP requirements for maintenance
C_s_: substrate concentration
K_s_: substrate saturation constant
