# Supplementary figures and images for: Growth-rate dependency of de novo resveratrol production in chemostat cultures of an engineered Saccharomyces cerevisiae strain
Source: Microb Cell Fact. 2015 Sep 14;14:133. doi: 10.1186/s12934-015-0321-6 (PMC4570684; doi:10.1186/s12934-015-0321-6)

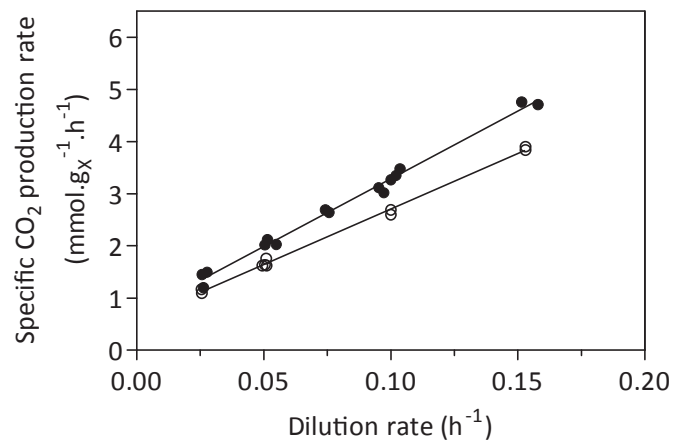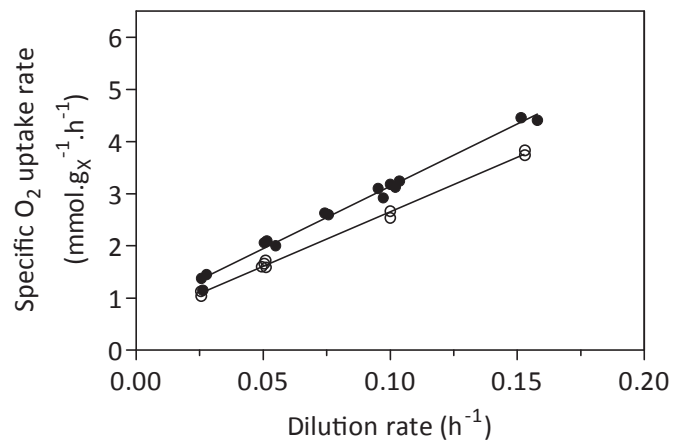

Supplement: Additional file 1: — Figure S1. Specific CO2 production and specific O2 uptake rates of the resveratrol producing S. cerevisiae strain FS09322 and the isogenic strain CEN.PK113-7D. Closed symbols indicate the resveratrol producing S. cerevisiae strain FS09322. Open symbols indicate isogenic strain CEN.PK113-7D. Each data point represents results from an individual chemostat. [file 12934_2015_321_MOESM1_ESM.pdf]

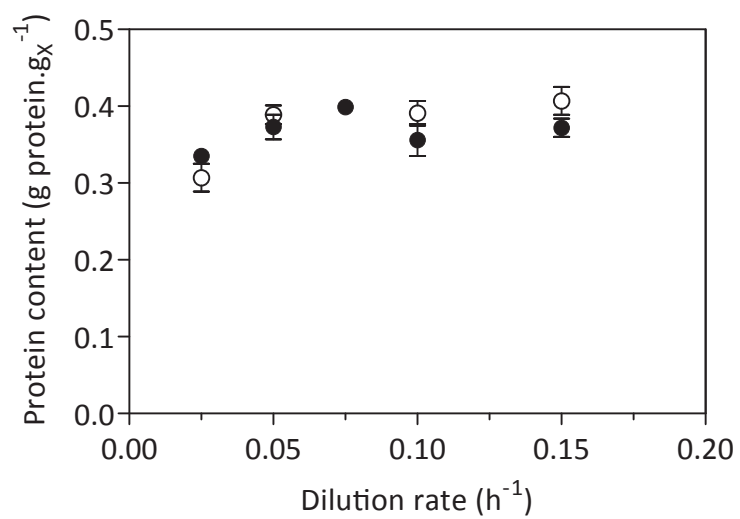

Supplement: Additional file 2: — Figure S2. Protein content of the resveratrol producing S. cerevisiae strain FS09322 and its isogenic strain CEN.PK113-7D. Open symbols indicate strain CEN.PK113-7D, close symbols indicate strain FS09322. The shown data represent the average and standard deviation of two independent culture replicates for each dilution rate and each strain. [file 12934_2015_321_MOESM2_ESM.pdf]

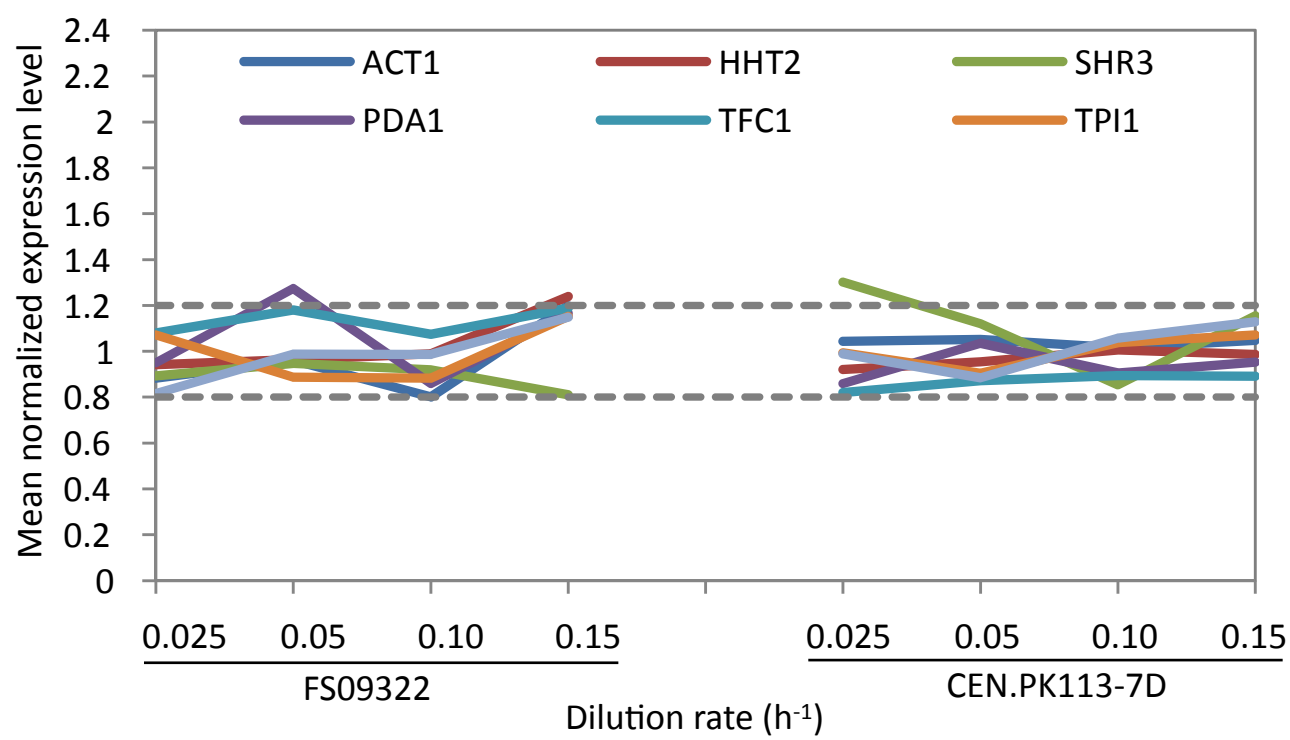

Supplement: Additional file 3: — Figure S3. Averaged normalized gene expression of housekeeping genes1 for S. cerevisiae strain FS09322 and CEN.PK113-7D. Dotted bars indicate 20 % variation around normalized expression. 1Teste MA, Duquenne M, Francois JM, Parrou JL: Validation of reference genes for quantitative expression analysis by real-time RT-PCR in Saccharomyces cerevisiae. BMC Mol Biol 2009, 10:99. [file 12934_2015_321_MOESM3_ESM.pdf]
