# Supplementary material for: Growth-rate dependency of de novo resveratrol production in chemostat cultures of an engineered Saccharomyces cerevisiae strain
Source: Microb Cell Fact. 2015 Sep 14;14:133. doi: 10.1186/s12934-015-0321-6 (PMC4570684; doi:10.1186/s12934-015-0321-6)
Supplement: Additional file 4: — Additional reactions for incorporation of resveratrol biosynthesis in the stoichiometric model. [file 12934_2015_321_MOESM4_ESM.docx]

**Additional reactions for incorporation of resveratrol biosynthesis in the stoichiometric model**

L-phenylalanine ammonia-lyase

1 H:cyt + 1 PHE:cyt <=> 1 Cin:cyt + 1 NH4:cyt

Cinnamate 4-hydroxylyase

1 Cin:cyt + 1 H:cyt + 1 NADPH:cyt + 1 O2:cyt <=> 1 Cou:cyt + 1 H2O:cyt + 1 NADP:cyt

Coumarate CoA ligase

2 ATP:cyt + 1 CoA:cyt + 1 Cou:cyt + 2 H:cyt + 1 H2O:cyt <=> 2 ADP:cyt + 1 CouCoA:cyt + 2 Pi:cyt

Reservatrol synthase

1 CouCoA:cyt + 3 MACoA:cyt <=> 4 CO2:cyt + 4 CoA:cyt + 1 H:cyt + 1 res:cyt

NAD glutamate dehydrogenase; Gdh2

1 H:cyt + 1 NADH:cyt + 1 NH4:cyt + 1 OGL:cyt <=> 1 GLM:cyt + 1 H2O:cyt + 1 NAD:cyt

NAD acetaldehyde dehydrogenase, Ald2/3

1 ACTAL:cyt + 1 H2O:cyt + 1 NADP:cyt <=> 1 ACT:cyt + 2 H:cyt + 1 NADPH:cyt

acetaldehyde ACTAL C2H4O

acetyl-CoA ACCoA C23H34N7O17P3S-4

ADP ADP C10H12N5O10P2-3

carbondioxide CO2 CO2

chorismate CHO C10H8O6-2

Cinnamate Cin C9H8O2

CoA CoA C21H32N7O16P3S-4

Coumarate Cou C9H8O3

CoumaroylCoA CouCoA C30H42N7O18P3S

glutamate GLM C5H8NO4-1

hydrogen H H+1

malonyl-CoA MACoA C24H33N7O19P3S-5

NAD NAD +1

NADH NADH H

NADP NADP +1

NADPH NADPH H

NH4 NH4 H4N+1

oxoglutarate OGL C5H4O5-2

oxygen O2 O2

phenylalanine PHE C9H11NO2

phosphate Pi HO4P-2

reservatrol res C14H12O3

water H2O H2O
